# Exploring *IL-10* and *NOS3* Genetic Variants as a Risk Factor for Neonatal Respiratory Distress Syndrome and Its Outcome

**DOI:** 10.3390/diagnostics15172259

**Published:** 2025-09-06

**Authors:** Mădălina Anciuc-Crauciuc, George-Andrei Crauciuc, Florin Tripon, Marta Simon, Manuela Camelia Cucerea, Claudia Violeta Bănescu

**Affiliations:** 1 Doctoral School of Medicine, Institution Organizing University Doctoral Studies (IOSUD), George Emil Palade University of Medicine, Pharmacy, Science, and Technology, 540142 Târgu Mureș, Romania; madalina.anciuc@umfst.ro (M.A.-C.); claudia.banescu@umfst.ro (C.V.B.); 2 Genetics Department, George Emil Palade University of Medicine, Pharmacy, Science, and Technology, 540142 Târgu Mureș, Romania; florin.tripon@umfst.ro; 3 Neonatology Department, George Emil Palade University of Medicine, Pharmacy, Science, and Technology, 540142 Târgu Mureș, Romania; marta.simon@umfst.ro (M.S.); manuela.cucerea@umfst.ro (M.C.C.); 4 Genetics Laboratory, Center for Advanced Medical and Pharmaceutical Research, George Emil Palade University of Medicine, Pharmacy, Science, and Technology, Gheorghe Marinescu 38, 540139 Târgu Mureș, Romania

**Keywords:** genetic variants, neonatal respiratory distress syndrome, *IL-10* gene, *NOS3* gene

## Abstract

**Background/Objective:** Neonatal respiratory distress syndrome (RDS) is a leading cause of morbidity and mortality in preterm infants. Interleukin-10 (IL-10) and endothelial nitric oxide synthase (eNOS, also known as *NOS3*) regulate inflammation and vascular tone, and genetic variants may influence the risk of RDS. To investigate the association between *IL-10* rs1800872 (c.-149+1984T>G), *IL-10* rs1800896 (c.-149+2474T>C), and *NOS3* rs2070744 (c.-149+1691C>T), *NOS3* rs1799983 (c.894T>G) variants and the risk of RDS in a Romanian cohort of preterm neonates. **Methods:** This case–control study included 340 preterm neonates (113 with RDS, 227 controls) born at <36 weeks of gestation. Genotyping was performed using TaqMan SNP assays. Logistic regression adjusted for gestational age and sex estimated odds ratios (ORs) and 95% confidence intervals (CIs). ROC analyses evaluated predictive performance. **Results:** No significant differences in genotype or allele distributions were observed between RDS and control groups for any variant. Haplotype analysis also revealed no association with RDS susceptibility or severity. *NOS3*:c.894T>G variant was associated with reduced risk of severe RDS after correction (adjusted *p* = 0.009), though survival analysis showed no significant genotype-specific effects. Epistatic genotype interaction was observed for the *IL-10* T/G + T/C, present only in RDS (*p* = 0.0026). ROC analysis revealed a clinical prediction of RDS (AUC = 0.996), while the addition of genetic variants improved discrimination for severity (AUC = 0.865; 95% CI: 0.773–0.957) and mortality (AUC = 0.913; 95% CI: 0.791–1.000). **Conclusions:** *IL-10* and *NOS3* variants were not individually associated with overall RDS susceptibility. The observed epistatic interactions and the potential protective effect of *NOS3*:c.894T>G against severe forms can suggest modulatory roles in disease progression. Larger, ethnically homogeneous cohorts are needed to confirm these findings and assess their potential for informing personalized care for neonates.

## 1. Introduction

Interleukin-10 (IL-10) was initially identified by Mosmann and colleagues as a cytokine synthesis inhibitory factor (CSIF), characterized by its capacity to suppress the activity of pro-inflammatory T helper 1 (Th1) cells [1]. Although initially identified as being secreted by Th2 cells, this cytokine is now recognized to be produced by a broad range of cell types, encompassing both immune and non-immune cells [1]. Functionally, IL-10 plays a crucial role in mediating anti-inflammatory and immunoregulatory responses, acting across various immune cell subsets, including specific populations of T cells, B cells, alternatively activated macrophages, and dendritic cells [2,3]. IL-10 is believed to exert a wide range of biological effects, extending beyond immune regulation to include roles in controlling blood vessel formation, maintaining vascular integrity, and regulating programmed cell death [4]. During pregnancy, it is produced by both trophoblastic and decidual tissues and appears to be essential for the proper development of the embryo. Insufficient levels of IL-10 have been associated with early pregnancy failure in both experimental animal models and human cases of recurrent miscarriage [5]. Furthermore, a lack of IL-10 during mid-gestation has been implicated in heightened inflammatory responses, contributing to preterm birth triggered by bacterial components in animal studies [6]. IL-10 is also a key regulator of immune activity in the developing lungs of newborns. By suppressing the release of pro-inflammatory molecules, such as tumor necrosis factor-alpha and interleukin-6, IL-10 helps reduce inflammation-related damage within the lung tissue. This function is especially vital in premature infants, where an excessive inflammatory response can lead to more severe lung injury and hinder the normal formation of alveoli [7,8]. However, elevated IL-10 levels in cord blood have been observed in preterm births and are associated with an increased risk of respiratory distress syndrome (RDS), highlighting the complex interplay between inflammatory regulation and disease pathogenesis. These findings may indicate a corrective response to prenatal or perinatal inflammatory stimuli rather than a direct protective effect, underscoring the multifaceted role of IL-10 in neonatal respiratory health [9,10,11,12].

Genetic variants in the *IL-10* gene, particularly the rs1800896 (c.-149+2474T>C) variant, have been associated with different levels of IL-10 expression, potentially influencing the risk of developing neonatal RDS [13]. The genotypes at this locus may be responsible for elevated IL-10 mRNA expression and enhanced anti-inflammatory effect [14].

Similarly, endothelial nitric oxide synthase (eNOS), encoded by the *NOS3* gene located on chromosome 7q35-36, plays a pivotal role in regulating pulmonary vascular tone and gas exchange through the production of nitric oxide (NO), a critical vasodilatory and signaling molecule. The gene spans 26 exons and harbors several variant loci, though only a subset has demonstrated functional relevance. Among these, the rs1799983 (c.894T>G), which involves a guanine-to-thymine substitution in exon 7 at nucleotide position 894, leads to an amino acid change from glutamic acid to aspartic acid. This alteration has been associated with decreased basal NO production and impaired endothelium-dependent vasodilation.

Disruptions in the eNOS-NO signaling axis have been implicated in the pathophysiology of various neonatal pulmonary conditions, including pulmonary hypertension and impaired oxygenation, both of which are relevant in the context of neonatal RDS [15]. Experimental models have demonstrated that eNOS deficiency can lead to abnormal lung development under mild postnatal hypoxia, characterized by reduced alveolarization and diminished vascular density—features that closely resemble those observed in bronchopulmonary dysplasia (BPD). Furthermore, genetic variants such as *NOS3*:c.894T>G and rs2070744 (*NOS3*:c.-149+1691C>T) have been associated with alterations in NO bioavailability and endothelial function. Some studies suggest that carriage of the variant allele at the *NOS3*:c.894T>G polymorphic locus may independently influence adverse pulmonary vascular and alveolar development in preterm neonates [16].

As allele frequencies and genotype effects may vary significantly across different ethnic and geographic backgrounds, studies have emphasized the need for population-specific investigations into genetic predispositions related to neonatal RDS over time. Despite growing evidence supporting the involvement of *IL-10* and *NOS3* variants in modulating inflammatory and vascular responses in neonates [13,14,17,18,19,20,21], data from Eastern European populations, particularly Romanian cohorts, remain limited. This study addresses this gap by analyzing the genotype and allele distributions of *IL-10* rs1800872 (c.-149+1984T>G), *IL-10*:c.-149+2474T>C, *NOS3*:c.-149+1691C>T, and *NOS3*:c.894T>G variants in a group of neonates diagnosed with RDS compared to a matched control group. By integrating molecular genotyping with clinical phenotypes, we aim to determine whether these variants play a measurable role in susceptibility to RDS in this population and contribute new insights to the global effort of understanding genetic influences on neonatal lung disease.

## 2. Materials and Methods

### 2.1. Ethical Consideration

In accordance with the ethical principles set forth in the Declaration of Helsinki, all research procedures were conducted following prior approval from the Ethics Committees of both the George Emil Palade University of Medicine, Pharmacy, Science and Technology of Târgu Mureș and the Emergency Clinical County Hospital of Târgu Mureș (renewed approval no. 23520, issued on 9 October 2023). Written informed consent was obtained from parents before enrolling any subject in the study.

### 2.2. Study Population

The study included 340 preterm singleton and twin neonates born between July 2020 and July 2025 at the Târgu Mureș Emergency Clinical County Hospital Maternity, a tertiary-level referral center. Eligible participants were live-born infants delivered before 36 weeks of gestation and admitted to the neonatal unit within the study period. All enrolled neonates were of white European descent.

Both groups were rigorously screened, and neonates were excluded if they were born to mothers with significant perinatal risk factors, such as premature rupture of membranes >18 h, clinical or histological chorioamnionitis, maternal infection, or exposure to medications known to affect neonatal respiratory outcomes. Additionally, patients with documented major congenital anomalies or missing clinical or genetic information were excluded from that specific analysis (complete-case analysis). The overall proportion of missing data was <5%, and thus the potential for bias due to case exclusion was considered minimal. No data imputation was performed. The final study cohort was divided based on the presence or absence of neonatal RDS. Of the 340 neonates, 113 were diagnosed with RDS, while 227 served as the comparison group, consisting of healthy preterm infants without clinical or radiological signs of RDS.

The reference standard for diagnosing neonatal RDS in our study was the European Consensus Guidelines on the Management of RDS. Diagnosis and inclusion in the RDS group required: (i) clinical evidence of respiratory distress within the first hours of life, assessed using the Silverman–Anderson score (0–3 mild, 4–6 moderate, >6 severe); (ii) the need for noninvasive ventilation or mechanical ventilation within the first 48 h of life; and (iii) characteristic chest X-ray findings consistent with at least Grade 2 RDS [22]. Supporting signs included tachypnea (>60 breaths/min), intercostal or subcostal retractions, nasal flaring, expiratory grunting, and a requirement for supplemental oxygen to maintain SpO_2_ ≥ 88% with FiO_2_ ≥ 0.3 [23,24,25]. None of the neonates received surfactant prior to the confirmation of RDS.

### 2.3. Sample Collection and DNA Extraction

Genomic deoxyribonucleic acid (DNA) was obtained from either peripheral venous blood or buccal epithelial cells, depending on the availability of the sample and the clinical context. For buccal cell collection, DNA/ribonucleic acid (RNA) Buccal Swabs—SK-2S ISOHELIX™ (Cell Projects Ltd., Kent, UK)—were employed in accordance with the manufacturer’s protocol. The swabs were designed for noninvasive sample collection and provided a reliable source of nucleic acids for downstream molecular analysis.

DNA extraction was subsequently carried out using the NucleoSpin^®^ QuickPure (Macherey-Nagel GmbH & Co., KG, Valencienner Str. 11, Düren, Germany), which ensures high-purity DNA suitable for polymerase chain reaction (PCR) and other molecular applications. The procedure involved cell lysis, binding of DNA to a silica membrane under chaotropic conditions, multiple wash steps to remove contaminants, and final elution in a low-salt buffer or nuclease-free water. The quality and concentration of the extracted DNA were assessed using spectrophotometric methods, and samples were stored at −20 °C until further analysis.

### 2.4. Selection of Variants

A total of four variants within the *IL-10* and *NOS3* genes with potential functional impact were selected using the publicly available database of the National Center for Biotechnology Information (NCBI) for single-nucleotide polymorphisms (http://www.ncbi.nlm.nih.gov/snp (accessed on 25 May 2025); Bethesda, MD, USA). The selection was based on three criteria: (i) previously reported associations with neonatal RDS and their potential functional relevance in inflammatory and vascular pathways, (ii) a minor allele frequency (MAF) greater than 0.05 in the European population, and (iii) genomic location either in the coding region resulting in a non-synonymous substitution or within regulatory elements. The panel included variants within the *IL-10* gene, c.-149+1984T>G and c.-149+2474T>C, and the *NOS3* gene, c.-149+1691C>T and c.894T>G. The selected variants are summarized in Table 1.

### 2.5. Genotyping

Genotyping of selected variants was performed using real-time polymerase chain reaction (qPCR) with TaqMan^®^ SNP Genotyping Assays (ThermoFisher Scientific, 168 Third Avenue, Waltham, MA, USA). Each reaction was carried out in a total volume of 20 µL, including 10 µL of TaqMan Genotyping Master Mix, 1 µL of the specific SNP assay mix (containing primers and fluorescently labeled probes), 20 ng of genomic DNA, and nuclease-free water. Thermal cycling conditions were set as follows: an initial denaturation at 95 °C for 10 min, followed by 40 cycles of denaturation at 95 °C for 15 s and annealing/extension at 60 °C for 1 min. Allelic discrimination was performed using the QuantStudio™ 5 Real-Time PCR System (Applied Biosystems, 850 Lincoln Centre Dr., Foster City, CA, USA), and results were analyzed using the accompanying software.

### 2.6. Molecular Analysis

Quality control measures included the use of negative controls (no-template controls) and repeat genotyping of 10% of the samples selected at random to ensure reproducibility. Genotype distributions were evaluated for Hardy–Weinberg equilibrium. Data were compiled for statistical association analysis to investigate the relationship between genetic variants and the presence of RDS in the study population.

### 2.7. Evaluation of Data

#### 2.7.1. Overview Statistics

Demographic profiles and clinical characteristics were summarized using basic statistical measures. Continuous variables were presented as means with standard deviations, while categorical variables were described through proportions and counts.

#### 2.7.2. Statistical Analysis

The primary outcome was the presence of neonatal RDS, analyzed in relation to *IL-10* and *NOS3* variants using chi-square and logistic regression. Secondary outcomes included severity, complications, and survival.

For this investigation, data processing and statistical evaluation were performed with SPSS software (version 20, Chicago, IL, USA). Relationships between genotype patterns and categorical parameters were examined using cross-tabulation, employing the chi-square method; Yates’ correction was applied when necessary. In instances where expected values fell below 5 in more than one cell, Fisher’s exact method was substituted. For variables following a normal distribution, comparisons were made using the independent samples *t*-test.

Odds ratios (ORs) were calculated to assess the relative risk or likelihood of developing neonatal RDS associated with specific genetic variants. The chi-square (χ^2^) test evaluated the conformity of allele distributions with Hardy–Weinberg equilibrium expectations. The potential predictive value of each variant for RDS was examined through univariate logistic regression analysis. Gestational age and biological sex were included as covariates in the regression model to account for their potential confounding effects on variant-associated outcomes. Statistical significance was defined as a *p*-value less than 0.05.

Haplotype configuration and its association with RDS-related variants were examined using Haplotype Analysis software version 1.05, provided by Georg-August-Universität Göttingen, Germany.

Discriminative performance was evaluated using binary logistic regression models based on genetic predictors, clinical variables, or their combination. Receiver operating characteristic (ROC) curves were generated, and the area under the curve (AUC) with 95% confidence intervals was calculated using the Hanley–McNeil method. The optimal cut-off was determined by the Youden index, and sensitivity, specificity, likelihood ratios, and predictive values (recalibrated to an internal clinical prevalence of 35%) were reported.

To ensure the study had sufficient power to detect significant associations, an a priori power analysis was conducted using G*Power software (version 3.1.9.6, Heinrich-Heine University, Düsseldorf, Germany). Assuming a minor allele frequency of 25%, an odds ratio of 2.0, a control-to-case ratio of 2:1, and a two-sided significance level (α = 0.05), the study achieved a statistical power of over 80% with a final cohort of 340 participants. The calculation was based on the methodology and formulas reported by Faul et al. [26] and Cohen [27].

To address the potential inflation of type I error due to multiple hypothesis testing, we applied both Bonferroni and Benjamini–Hochberg (FDR) corrections across all genotype-phenotype association models.

## 3. Results

### 3.1. Patient Demographics and Clinical Parameters

Among 340 neonates analyzed, gestational age, birth weight, gender, and maternal age did not differ between groups (all *p* > 0.2). RDS cases were less likely to have received adequate antenatal care (36.3% vs. 59.0%, *p* < 0.001) and were more often delivered by cesarean section (60.2% vs. 44.9%, *p* = 0.0113). Clinically, they had lower Apgar scores at 1 and 5 min (both *p* < 0.0001), longer NICU stays (19.5 vs. 9 days, *p* < 0.0001), and a higher incidence of PDA (14.1% vs. 2.6%, *p* < 0.001). Mortality occurred in 8.8% of RDS neonates (*p* < 0.001). Fatal cases showed poor treatment response despite repeated surfactant therapy, with no evidence of infection or remarkable findings on echocardiography. A comprehensive summary of clinical and demographic characteristics for all study participants is presented in Table 2.

### 3.2. Analysis of Genotypic and Allelic Distributions of IL-10 and NOS3 Variants in Relation to RDS Risk

Genotype distributions of *IL-10*:c.-149+1984T>G, *IL-10*:c.-149+2474T>C, *NOS3*:c.-149+1691C>T, and *NOS3*:c.894T>G were compared between RDS and controls using codominant, dominant, recessive, and overdominant models (Table 3). In the primary analysis, no significant associations were observed for the *IL-10* variants (all *p* > 0.05). For *NOS3*:c.-149+1691C>T, an association was detected in the overdominant model (*p* = 0.03), but this lost significance after adjustment for gestational age in multivariate logistic regression and did not withstand multiple testing correction (Bonferroni/FDR). Multivariate analyses adjusted for gestational age and birth weight confirmed no significant associations between any tested genotypes and RDS (all *p* > 0.05).

Allele frequency and statistical analysis were performed for the investigated variants, comparing the RDS and control groups. Additionally, a comparison with the frequency of alleles in different populations was performed (Table 4). No statistically significant association was identified between the investigated variants and RDS risk. Comparing the allele frequency for *IL-10*:c.-149+1984T>G, the overall results of our study was 65%, which is slightly lower than the frequency reported in the European population (76%) and a more pronounced difference was observed for *IL-10*:c.-149+2474T>C, where the C allele frequency in our cohort was lower than in Europeans (48%) but substantially higher than in the East Asian population (5%).

The influence of genetic variants on RDS severity was assessed by contingency analysis and ordinal logistic regression. A significant association was observed for *NOS3*:c.894T>G, where the G allele was linked to a reduced risk of severe RDS (Bonferroni- and FDR-adjusted *p* = 0.009). No other variants showed significant associations with severity (all *p* > 0.05).

To further assess predictive capacity, ROC analyses were performed for models incorporating genetic predictors, clinical variables, and their association.

**Figure 1:**
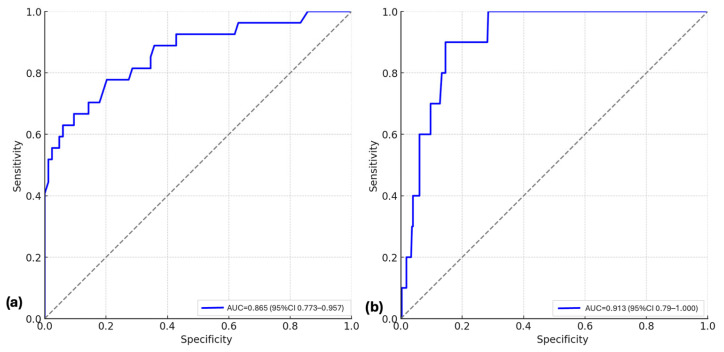
ROC curves for the combined genetic and clinical models: (**a**) ROC curve for predicting severe versus non-severe RDS among affected neonates; (**b**) ROC curve for predicting mortality. The diagonal dashed line represents the reference line of no discrimination (AUC = 0.5).

For RDS severity, the combined model achieved an AUC of 0.865 (95% CI, 0.773–0.957), outperforming the clinical-only model (AUC 0.810, 95% CI 0.705–0.915) and the genetic-only model (AUC 0.704, 95% CI 0.584–0.825). At the optimal threshold, sensitivity was 0.78 and specificity 0.80, with recalibrated values of PPV 67.4% and NPV 41.6% (Figure 1a). For mortality, the combined model also demonstrated strong discrimination (AUC 0.913, 95% CI 0.791–1.000), which was higher than both the clinical-only model (AUC 0.606, 95% CI 0.448–0.763) and the genetic-only model (AUC 0.723, 95% CI 0.572–0.874). At the Youden-derived threshold, sensitivity was 0.87, and specificity was 0.57, with recalibrated PPV 52.0% and NPV 34.7% (Figure 1b).

### 3.3. Haplotype and Linkage Disequilibrium Analysis of IL-10 and NOS3 Variants in Neonates with RDS

Haplotype analysis of *IL-10* and *NOS3* variants was performed to evaluate the potential association with RDS and clinical outcomes. In *IL-10*, the most frequent haplotype of c.-149+1984T>G and c.-149+2474T>C was GT, with an overall frequency of 38% (38.8% in controls and 37.2% in RDS groups). For *NOS3*, the most frequent haplotype of c.-149+1691C>T and c.894T>G was TG, with a total frequency of 26% (22% in the control group and 30% in the RDS group). There were no statistically significant differences in haplotype frequencies between the cases and controls (*p* > 0.05), suggesting no association with RDS.

Moreover, we investigated whether the distributions of *IL-10* and *NOS3* haplotypes were associated with RDS severity among different gestational age subgroups. Chi-square analysis revealed no statistically significant associations in either the extremely/very premature group (*IL-10*: *p* = 0.78; *NOS3*: *p* = 0.81) or the moderately premature group (*IL-10*: *p* = 0.23; *NOS3*: *p* = 0.09). Although not reaching statistical significance, the observed trend for *NOS3* haplotypes in moderately premature infants may indicate a potential biological relevance, warranting validation in a larger cohort.

Linkage disequilibrium (LD) was analyzed for all variants investigated in the RDS and the control group. *NOS3*:c.-149+1691C>T and *NOS3*:c.894T>G showed the strongest linkage with D’ value of 0.32 and r^2^ 0.16, indicating a moderate degree of allelic correlation. In contrast, the *IL-10*:c.-149+1984T>G and *IL-10*:c.-149+2474T>C showed a weak linkage, with D’ and r^2^ values of 0.12 and 0.054, suggesting a large independent inheritance pattern. Moreover, no substantial LD was observed between polymorphisms of *IL-10* and *NOS3*, supporting the assumption that the loci are inherited independently. These findings suggest that the *IL-10* and *NOS3* variants are unlikely to influence RDS susceptibility through common inheritance patterns or linked genetic mechanisms.

### 3.4. Epistatic and Environmental Modifiers of Genetic Risk in Neonatal RDS

**Figure 2:**
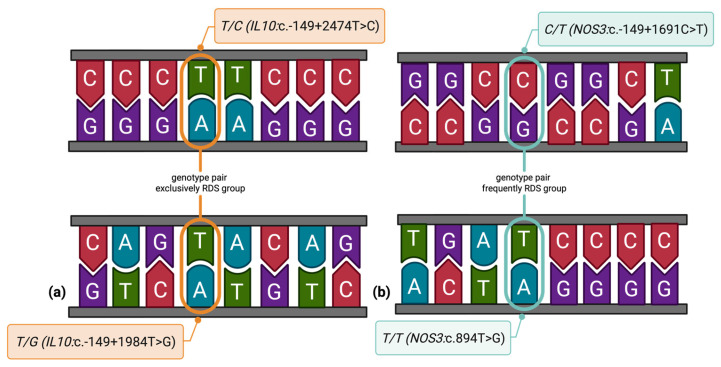
Epistatic effects of *IL-10* and *NOS3* variants in neonatal RDS. Schematic representation of nucleotide substitutions in the *IL-10* and *NOS3* genes. (**a**) the genotype pair T/G (c.-149+1984T>G) and T/C (c.-149+2474T>C) in *IL-10* was exclusively observed in neonates with RDS (*p* = 0.0026); (**b**) the genotype pair C/T (c.-149+1691C>T) and T/T (c.894T>G) in *NOS3* occurred more frequently among RDS cases than in controls (*p* = 0.0482).

To explore potential epistatic effects between *IL-10* and *NOS3* variants, we conducted a variant-to-variant interaction analysis using combined genotype data. Among all tested variant pairs, two genotype combinations demonstrated statistically significant associations with RDS (Figure 2), highlighting potential epistatic interactions between *IL-10* and *NOS3* variants that may contribute to RDS susceptibility.

Gene–environment interaction analysis was attempted through a preliminary approach, but stratified genotype distributions by different environmental factors (like gender, gestational age, antenatal care, and necessity of mechanical ventilation) showed limited variability, precluding formal statistical testing. This highlights the need for larger, more genetically diverse cohorts to evaluate potential gene–environment effects in neonatal RDS. Stratified analysis by surfactant treatment revealed no significant association between *IL-10*:c.-149+2474T>C genotype and RDS among untreated neonates (*p* = 1.00), while the treated subgroup lacked sufficient genotype variability for valid inference.

### 3.5. Genetic Associations with Neonatal Complications

To further investigate the relationship between genetic factors and the risk of BPD, a multivariate logistic regression analysis was performed, including all investigated variants. The analysis did not reveal any statistically significant associations between the investigated *IL-10* and *NOS3* variants (*p* > 0.05).

No statistically significant associations were identified between any of the investigated *IL-10* and *NOS3* genotypes and BPD, or other neonatal complications analyzed, including RDS requiring mechanical ventilation, PDA, early-onset sepsis, intraventricular hemorrhage, pulmonary hemorrhage, ROP greater than stage 2, prolonged NICU stay, or death.

### 3.6. Genotype-Based Kaplan–Meier Survival Analysis

**Figure 3:**
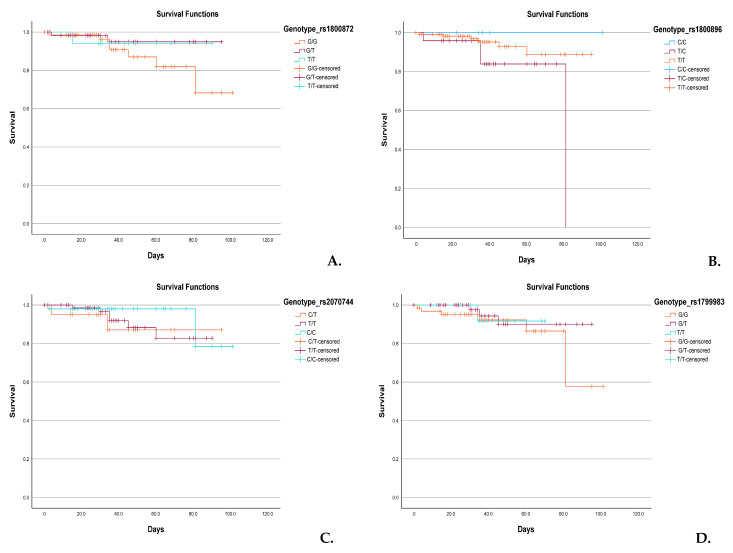
Kaplan–Meier Survival Curves Stratified by *IL-10* and *NOS3* Genotypes. (**A**) *IL-10*:c.-149+1984T>G, (**B**) *IL-10*:c.-149+2474T>C, (**C**) *NOS3*:c.-149+1691C>T, and (**D**) *NOS3*:c.894T>G. Survival probabilities are shown for each genotype group over time (days). The curves illustrate the survival during hospitalization, comparing all three genotypes of the investigated variants. Survival rates were compared using the log-rank test, and no statistically significant differences were observed between genotype groups. Censored cases are indicated by tick marks.

Kaplan–Meier survival analyses were conducted to assess the potential impact of specific *IL-10* and *NOS3* variants on neonatal survival (Figure 3). For *IL-10*:c.-149+1984T>G (Figure 3A), neonates homozygous for the G allele (G/G) showed a modest reduction in survival probability beyond the first month compared to heterozygous (G/T) and homozygous T (T/T) carriers. Similarly, in *IL-10*:c.-149+2474T>C (Figure 3B), a notable decline in survival was observed in T/T individuals around day 80, while C/C and C/T groups maintained higher survival rates. Analysis of *NOS3*:c.-149+1691C>T (Figure 3C) indicated slightly reduced survival among neonates with the C/C genotype, although the difference between groups was not statistically significant. In *NOS3*:c.894T>G (Figure 3D), neonates with the G/G genotype showed a survival drop after 80 days, while T/T and G/T groups had more favorable survival trajectories.

Although none of the genotype-specific survival differences reached statistical significance (log-rank test, *p* > 0.05), the observed trends may suggest genotype-related modulation of disease severity or complications influencing survival in neonates with RDS.

## 4. Discussion

This study aims to investigate the association between the *IL-10*:c.-149+1984T>G, *IL-10*:c.-149+2474T>C, *NOS3*:c.-149+1691C>T, and *NOS3*:c.894T>G gene variants and susceptibility to RDS, in order to determine whether these variants play a measurable role in the development of RDS in this population and contribute new insights to the global effort of understanding genetic influences on neonatal lung disease. We utilized the latest allele annotations, based on the NCBI and Ensembl genome browsers. In earlier studies, allele designations were often reversed [13,14,16]. Currently, for *IL-10*:c.-149+1984T>G, the G allele is ancestral, whereas the T allele serves as the reference [28]. For *IL-10*:c.-149+2474T>C, the T allele is both ancestral and reference [29]. In *NOS3*:c.-149+1691C>T, the C allele is both ancestral and reference, while, in *NOS3*:c.894T>G, G is ancestral, but T is the reference [30,31].

The diagnosis of neonatal RDS currently relies on a combination of perinatal history, clinical presentation, radiographic findings, and blood gas analysis. Chest X-ray, however, has limited sensitivity (~35%) and modest diagnostic accuracy (~60%) in differentiating RDS from other neonatal respiratory disorders [32,33]. Lung ultrasound (LUS) offers higher specificity and avoids radiation but remains limited in availability and does not address genetic susceptibility [34,35]. Similarly, PCR and immunoassays can detect infectious or inflammatory contributors but cannot predict individual risk or disease progression. As uniform treatment strategies overlook heterogeneity among preterm infants, precision medicine (integrating genetic predisposition, clinical characteristics, and biomarkers) may improve risk stratification and management [32]. Genetic variants, including *IL-10* and *NOS3*, may refine susceptibility assessment beyond current diagnostic tools [13,33].

The biological plausibility of this approach is supported by experimental and clinical evidence. IL-10 regulates pulmonary inflammation, with reduced expression linked to cytokine-driven alveolar injury in preterm infants [9]. *NOS3* is essential for neonatal cardiopulmonary transition; eNOS-deficient mice develop impaired alveolarization, pulmonary hypertension, and fatal respiratory distress [36]. Variants affecting these pathways may therefore influence RDS susceptibility and severity, providing a mechanistic rationale for their study in neonatal cohorts.

### 4.1. IL-10 Variants and Their Role in RDS Susceptibility

IL-10 is a pivotal immunoregulatory cytokine secreted by diverse immune cells, including T and B lymphocytes, NK cells, antigen-presenting cells, mast cells, and granulocytes [37,38]. It attenuates inflammation and regulates the differentiation and proliferation of immune cells. Systemic release of IL-10 also provides neuroendocrine-mediated protection against excessive inflammation during acute stress. Conversely, relative IL-10 deficiency is observed in disorders dominated by type 1 cytokine responses [39]. Even low levels of proinflammatory cytokines during pregnancy may reduce the risk of RDS in preterm infants [40]. Although the precise mechanisms regulating *IL-10* remain unclear, genetic factors appear central [41].

Evidence linking *IL-10* variants to neonatal lung disease is limited; however, differences in IL-10 expression have been described in preterm infants with RDS, particularly in relation to BPD [17,18,42]. In our cohort, no significant associations were found between *IL-10*:c.-149+1984T>G and *IL-10*:c.-149+2474T>C variants and either RDS susceptibility or survival. Nevertheless, functional studies suggest regulatory relevance. The c.-149+1984T>G variant, first reported by Hobbs et al. [43], interferes with promoter regulation and may alter transcriptional output [44,45]. Capasso et al. found no association with RDS but demonstrated that the c.-149+2474T>C reference allele correlated with significantly higher *IL-10* mRNA expression in EBV-transformed lymphoblastoid cells [14]. Mechanistically, the variant allele enhances expression at mRNA and protein levels, whereas the reference allele increases PU.1 binding, suppressing transcription [46,47]. This differential regulation may influence the availability of IL-10 in the immature lung.

Clinically, some studies suggest protective effects of the *IL-10*:c.-149+2474T>C reference allele (OR = 0.48; 95% CI, 0.24–0.95; *p* = 0.03). Its higher frequency among infants without RDS implies a role in reducing inflammatory burden [14]. In contrast, the variant allele has been associated with diminished IL-10 levels and adverse outcomes, including BPD and mortality, in very low-birth-weight infants [18], as well as increased vulnerability to RDS [19]. Such findings suggest that reduced IL-10 synthesis may amplify inflammatory responses and increase disease risk.

IL-10 is detectable in bronchoalveolar lavage fluid of ventilated preterm infants with RDS, particularly in the first five days of life [48]. Exogenous IL-10 supplementation reduces proinflammatory cytokines and mortality, whereas antibody-mediated IL-10 blockade worsens inflammation and survival [49].

Our study does not contradict this hypothesis, but it may reflect population-specific effects or sample size limitations that preclude the detection of modest influences.

### 4.2. NOS3 Gene Variants and Neonatal Pulmonary Outcomes

NO regulates apoptosis, inflammation, and endothelial barrier function. Its production is mediated by the NOS enzyme family, comprising neuronal (NOS1), inducible (NOS2), and NOS3 isoforms [50], with eNOS representing the main physiological source of vascular NO [51]. Variants in *NOS3*, particularly c.894T>G and c.-149+1691C>T, have been associated with adverse neonatal outcomes, including impaired cerebral blood flow and increased risk of IVH [52], ROP requiring treatment [53], perinatal HIE (G allele of c.894T>G), and PPHN (T/C genotype and C allele of c.-149+1691C>T) [54]. NO also plays a pivotal role in postnatal pulmonary transition by lowering pulmonary vascular resistance and optimizing ventilation–perfusion matching [55]. In experimental models, NOS3 deficiency leads to profound lung abnormalities, respiratory failure, and early mortality [36], highlighting its role in pulmonary development, although its specific contribution to neonatal RDS remains incompletely defined [55].

We hypothesized that *NOS3* variants might predispose preterm infants to maladaptive vascular responses, thereby increasing their susceptibility to RDS. However, no significant differences in genotype or allele frequencies were observed between RDS and control groups, consistent with studies in Chinese Han and Turkish populations [20,21,56]. When stratified by gestational age, higher frequencies of the homozygous reference genotype and allele were observed in RDS infants, whereas heterozygous and variant alleles were less common [20]. Sex-specific distributions were also noted, with the variant allele more frequent in males [20,21]. In our cohort, the *NOS3*:c.894T>G variant was associated with a reduced risk of severe RDS, suggesting that it may influence disease severity rather than onset. This association may be biologically explained if serum protein levels are not reduced (or are increased) or if gene expression is elevated or unaffected. The effect was modest, and our limited sample size precludes firm conclusions about predictive use. This finding aligns with Shen et al., who reported a protective effect of the homozygous reference genotype in very preterm, low-birth-weight infants; however, it contrasts with Turkish cohorts, where no association was found [16,21]. Discrepancies in database annotations (T as reference vs. ancestral G allele) may contribute to apparent inconsistencies across studies.

Haplotype and LD analyses did not reveal significant associations: *IL-10* (TC) and *NOS3* (TG) haplotypes were similarly distributed between groups, and weak LD indicated independent segregation of alleles [14]. Multivariate regression, adjusted for gestational age and birth weight, confirmed that none of the variants independently predicted RDS, although some genotype combinations (T/G and T/C in *IL-10*) appeared enriched among affected neonates, suggesting that their role is modulatory rather than predictive. 

Collectively, these findings suggest that, while *NOS3* and *IL-10* variants may contribute to vascular and immune mechanisms relevant to RDS, their individual effects are likely modest within the multifactorial etiology of the disease. When evaluating the contribution of clinical and genetic predictors to RDS outcomes, clinical factors alone demonstrated high discrimination for disease occurrence, whereas the addition of *IL-10* and *NOS3* variants improved the discrimination of disease severity and mortality. These findings highlight the potential of integrating genetic information for risk stratification, although confirmation in larger, independent cohorts is required.

### 4.3. Variant-to-Variant Interactions Between IL-10 and NOS3 Genes

The gene–gene interaction analysis identified two significant genotype combinations potentially associated with an increased risk of neonatal RDS. The genotype pair *T/G* (*IL-10*:c.-149+1984T>G) and *T/C* (*IL-10*:c.-149+2474T>C), both located within the *IL-10* regulatory region, were exclusively present in neonates with RDS and absent in the control group, reaching statistical significance. This exclusive clustering in affected neonates may reflect a synergistic risk effect of regulatory variants in the IL-10 locus, which has not been previously reported in European or Asian cohorts. Furthermore, the genotype pair *C/T* (*NOS3*:c.-149+1691C>T) and *T/T* (*NOS3*:c.894T>G), which includes functional variants within the *NOS3* gene, showed a significant enrichment in RDS cases. Given the role of *NOS3* in endothelial function and pulmonary vasoregulation, such combinations may alter nitric oxide availability and contribute to the pathophysiology of RDS. Although these findings should be interpreted cautiously due to limited subgroup sizes and lack of significance in logistic regression models, they offer preliminary evidence that interactions between *IL-10* and *NOS3* variants could influence disease susceptibility more strongly than individual variants alone. Further functional studies and replication in larger cohorts are essential to confirm these epistatic effects and to clarify their biological implications in neonatal lung development and inflammatory response.

### 4.4. In Silico Analysis of the Investigated Variants

The variants c.-149+1984T>G and c.-149+2474T>C, although located within an extended intronic region of the *IL-19* gene (NM_153758.5), are functionally positioned in the upstream regulatory region of *IL-10* and may influence its expression profile.

According to multiple in silico prediction tools, including CADD, RegulomeDB, and HaploReg, both variants are situated in genomic regions with epigenetic marks indicative of regulatory activity. They are predicted to interfere with transcription factor binding motifs involved in immune regulation, including those of NF-κB and STAT. Expression quantitative trait locus (eQTL) and data further support their association with altered IL-10 transcript levels in immune tissues. These findings are particularly relevant in the context of neonatal RDS, where insufficient anti-inflammatory signaling—potentially modulated by these variants—may contribute to increased disease susceptibility and severity in preterm infants. Similarly, the *NOS3*:c.-149+1691C>T and *NOS3*:c.894T>G variants may affect nitric oxide availability. The C allele of *NOS3*:c.-149+1691C>T is known to decrease eNOS promoter activity, while the T allele of *NOS3*:c.894T>G results in a structural change that can destabilize the enzyme [14,15]. These effects could impair endothelial function and pulmonary vasodilation, mechanisms that are particularly important in the pathophysiology of neonatal RDS. Overall, these findings support the hypothesis that genetic variation in *IL-10* and *NOS3* may modulate susceptibility and disease severity in affected newborns, suggesting potential functional implications relevant to neonatal RDS.

### 4.5. Population-Specific Context

Allele frequencies and genotype–phenotype relationships often vary across ethnic groups, which may explain conflicting results in the literature. While studies from Western Europe and Asia have reported both protective and risk-associated alleles for *IL-10* and *NOS3* variants, data from Eastern Europe remain sparse. Our study is among the first to explore these variants in a Romanian neonatal cohort, contributing novel insights into the genetic basis of RDS in this underrepresented population.

The nonassociation observed in our sample may reflect a genuine absence of effect or may be due to population-specific genetic structures, environmental exposures, or healthcare-related factors determining outcomes. These findings underscore the importance of conducting individualized genetic studies before extrapolating data across populations.

### 4.6. Clinical Implications and Future Directions

Our results did not provide evidence for a predictive function of the studied variants, but knowledge of the genetic factors of RDS remains critical for advancing personalized neonatal care. From a mechanistic standpoint, reduced IL-10 transcription linked to promoter polymorphisms may impair the anti-inflammatory capacity of the preterm lung, favoring cytokine-mediated alveolar injury and progression to severe RDS, as described in cord blood and pulmonary cell studies [9]. Likewise, *NOS3* variants that alter nitric oxide bioavailability can influence pulmonary vascular resistance and alveolar growth; experimental eNOS deficiency results in pulmonary hypertension, defective alveolarization, and neonatal respiratory failure [51,55]. These data support our observation that *IL-10* variants may cluster in RDS cases and that *NOS3*:c.894T>G carriers experience less severe disease. While unlikely to replace imaging-based diagnosis, such variants may refine severity prediction and help identify infants who would benefit from closer monitoring or adjunctive therapies, such as the targeted use of anti-inflammatory or NO-donor therapies. Given that inhaled nitric oxide (iNO) is already used in the management of conditions such as pulmonary hypertension, BPD, and hypoxemic respiratory failure, its primary benefit compared to conventional therapies is its ability to selectively dilate the pulmonary vasculature, leading to improved oxygenation [57,58,59,60].

Future studies should prioritize multicenter designs with larger sample sizes and integrate genetic data with transcriptomic, proteomic, and clinical parameters to enhance understanding of the disease. Such integrative approaches may help clarify the complex pathophysiology of RDS and identify genetic signatures that confer risk or resilience in vulnerable neonates. The assessment of serum protein levels and gene expression, along with the expansion of the study cohort, represents a future perspective for upcoming research projects. The absence of such comprehensive, diverse datasets represents a limitation of the present study.

Despite excluding major perinatal risk factors and adjusting for gestational age and birth weight, residual confounding (e.g., maternal factors, neonatal comorbidities) cannot be excluded. Selection and referral bias may arise from the single-center tertiary setting, while verification bias may occur from variability in RDS diagnosis. These aspects may have influenced the observed associations and should be taken into consideration when interpreting the results.

## 5. Conclusions

Although the primary outcome analysis showed no significant association between *IL-10* and *NOS3* variants and overall risk of RDS, the biological relevance of these genes in pulmonary inflammation and vasoregulation justifies further investigation. Secondary analyses identified significant associations between *IL-10*:T/G|T/C and *NOS3*:C/T|T/T genotype pairs and increased RDS risk, indicating possible epistatic effects. Additionally, a possible protective effect of the *NOS3*:c.894T>G variant against severe RDS suggests a modulatory role in disease progression. Predictive models incorporating these variants showed enhanced discrimination capacity compared to models using clinical factors alone. These exploratory findings warrant validation in larger, multicenter studies integrating genetic, clinical, and environmental data to clarify the potential protective or predisposing roles of these variants in neonatal respiratory outcomes.

## Figures and Tables

**Table 1:** Variants selected for the study.

Gene	NCBI dbSNP ID	Variant Localization	MAF *
*IL-10*	rs1800872	c.-149+1984T>G	0.49
*IL-10*	rs1800896	c.-149+2474T>C	0.27
*NOS3*	rs2070744	c.-149+1691C>T	0.40
*NOS3*	rs1799983	c.894T>G (p.Glu298Asp)	0.24

*—Minor allele frequency (MAF) in the European population (http://www.ncbi.nlm.nih.gov/snp (accessed on 25 May 2025)).

**Table 2:** Clinical and demographic characteristics for all study participants.

Variables	RDS Patients *n* = 113	Controls (Without RDS) *n* = 227	*p*-Values	OR; 95% IC
Gestational age (weeks): mean ± SD	31 ± 1.46	31 ± 1.57	1.0 *	0.66; 0.13–3.34
Gender: *n* (%)			0.8016 ^‡^	0.91; 0.56–1.46
Female	39 (34.51)	83 (36.56)		
Male	74 (65.48)	144 (63.43)		
Birth weight (g): mean ± SD	1476.44 ± 467.48	1504.24 ± 261.25	0.5574 *	1.12; 0.71–1.76
Preterm labor, *n* (%)	63 (55.75)	115 (50.66)	0.4412 ^‡^	1.23; 0.78–1.93
Singleton pregnancy, *n* (%)	86 (76.10)	197 (86.78)	0.0199 ^‡^	0.49; 0.27–0.86
Antenatal care, *n* (%)	41 (36.28)	134 (59.03)	0.0001 ^‡^	0.40; 0.25–0.63
Mother’s age, mean ± SD	29.7 ± 6.43	30.5 ± 4.78	0.2431 *	1.23; 0.69–2.19
Delivery mode, *n* (%)			0.0113 ^‡^	0.54; 0.34–0.85
Spontaneous	45 (39.82)	125 (55.06)		
C-section	68 (60.17)	102 (44.93)		
Delivery in a tertiary center, *n* (%)	87 (76.99)	188 (82.81)	0.2539 ^‡^	0.69; 0.40–1.21
Apgar Score, mean				
1 min	6	7	<0.0001 *	2.21; 1.37–3.56
5 min	7	8	<0.0001 *	2.20; 1.39–3.50
Surfactant use, *n* (%)	62 (54.86)	-		
LISA	36 (31.85)	-		
INSURE	7 (6.19)	-		
Standard	19 (16.81)	-		
Need for subsequent surfactant doses, *n* (%)	11 (9.73)	-		
Duration of non-invasive ventilation, hours (mean)	297.4 ± 138.9	-		
Duration of MV, hours (mean)	169.3 ± 35.8	-		
Chronic lung disease, *n* (%)	21 (18.58)	-		
PDA: *n* (%)	16 (14.15)	6 (2.64)	0.0001 ^†^	6.08; 2.31–15.99
Pulmonary hemorrhage, *n* (%)	6 (5.30)	-		
NICU days, mean ± SD	19.5 ± 6.3	-		
Deaths, *n* (%)	10 (8.84)	-		

Note: *n*—number; LISA—less invasive surfactant administration; INSURE—intubation–surfactant administration–extubation; MV—mechanical ventilation; PDA—patent ductus arteriosus; NICU—neonatal intensive care unit; *p*-values were obtained from generalized linear models; significant results were considered when *p*-values < 0.05; * *t*-test; ^‡^ χ^2^-test; ^†^ Fisher’s exact test.

**Table 3:** Genotype distribution and associations with RDS.

Variants	Gene Models	Genotypes	Controls (%)	RDS Cases (%)	OR (% 95)	*p* Value	OR (% 95) *	*p* Value *
*IL-10*:c.-149+1984T>G	Codominant	TT	37 (16)	16 (14)	-	-		
		TG	91 (40)	47 (42)	1.19 (0.60–2.36)	0.73	1.63 (0.31–8.38)	0.55
		GG	99 (44)	50 (44)	1.16 (0.59–2.30)	0.72	1.95 (0.18–2.70)	0.57
	Dominant	TT	37 (16)	16 (14)	-	-	-	-
		TG + GG	190 (84)	97 (86)	1.18 (0.62–2.22)	0.63	0.66 (0.07–5.94)	0.71
	Recessive	TT + TG	128 (56)	63 (56)	-	-	-	-
		GG	99 (44)	50 (44)	1.02 (0.65–1.61)	1.00	0.58 (0.12–2.74)	0.49
	Overdominant	TT + GG	136 (60)	66 (58)	-	-	-	-
		TG	91 (40)	47 (42)	1.06 (0.67–1.68)	0.81	1.37 (0.30–6.23)	0.41
*IL-10*:c.-149+2474T>C	Codominant	TT	181 (80)	86 (76)	-	-	-	-
		TC	43 (19)	22 (20)	1.07 (0.60–1.91)	0.88	0.88 (0.29–12.3)	0.95
		CC	3 (1)	5 (5)	3.50 (0.81–15.02)	0.12	0.52 (0.34–13.4)	0.75
	Dominant	TT	181 (80)	86 (76)	-	-	-	-
		TC + CC	46 (20)	27 (25)	1.23(0.71–2.12)	0.48	1.71 (0.28–3.22)	0.55
	Recessive	TT + TC	224 (99)	108 (96)	-	-	-	-
		CC	3 (1)	5 (5)	3.45 (0.81–14.73)	0.12	2.84 (0.64–10.24)	0.55
	Overdominant	TT + CC	184 (81)	91 (81)	-	-	-	-
		TC	43 (19)	22 (20)	1.03 (0.58–1.83)	1.00	1.62 (0.24–3.94)	0.49
*NOS3*:c.-149+1691C>T	Codominant	CC	44 (19)	17 (15)	-	-	-	-
		CT	86 (38)	57 (50)	1.71 (0.89–3.29)	0.11	3.70 (0.44–6.54)	0.22
		TT	97 (43)	39 (35)	1.04 (0.53–1.03)	1.00	2.57 (0.28–5.42)	0.40
	Dominant	CC	44 (19)	17 (15)	-	-	-	-
		CT + TT	183 (81)	96 (85)	1.35 (0.73–2.50)	0.37	3.18 (0.43–6.34)	0.25
	Recessive	CC + CT	130 (57)	74 (65)	-	-	-	-
		TT	97 (43)	39 (35)	0.70 (0.44–1.12)	0.15	3.18 (0.43–7.23)	0.25
	Overdominant	CC + TT	141 (62)	56 (50)	-	-	-	-
		CT	86 (38)	57 (50)	1.66 (1.05–2.64)	0.03	1.95 (0.42–6.98)	0.38
*NOS3*:c.894T>G	Codominant	TT	27 (12)	15 (14)	-	-	-	-
		TG	95 (42)	49 (43)	0.92 (0.45–1.90)	0.85	0.96 (0.25–3.22)	0.96
		GG	104 (46)	49 (43)	0.84 (0.41–1.73)	0.71	0.41 (0.23–3.58)	0.42
	Dominant	TT	27 (12)	15 (14)	-	-	-	-
		TG + GG	199 (88)	98 (86)	0.88 (0.45–1.74)	0.72	1.38 (2.64–8.31)	0.39
	Recessive	TT + TG	122 (54)	64 (57)	-	-	-	-
		GG	104 (46)	49 (43)	0.89 (0.56–1.41)	0.72	1.39 (0.32–1.72)	0.39
	Overdominant	TT + GG	131 (58)	64 (86)	-	-	-	-
		TG	95 (42)	49 (43)	1.05 (0.66–1.66)	0.81	1.22 (0.36–2.32)	0.79

Note: OR—odds ratio; 95% CI—95% confidence interval; * adjusted for gestational age regression analysis; *p*-values obtained from generalized linear models with a binomial distribution. Significant results were considered when *p*-values < 0.05.

**Table 4:** Allele distribution and statistical associations with RDS.

Variants	Variant Alleles	Overall Variant Allele Frequency/European Variant Allele Frequency/East Asian Allele Frequency (gnomAD v4.1.0)	Frequency of Variant Allele in Control Group (%)	Frequency of Variant Allele in RDS Group (%)	OR (% 95 CI) *	*p* Value
*IL-10*:c.-149+1984T>G	G	68/76/31	289 (64)	147 (65)	1.06 (0.76–1.48)	0.73
*IL-10*:c.-149+2474T>C	C	40/48/5	49 (11)	32 (14)	1.36 (0.84–2.19)	0.21
*NOS3*:c.-149+1691C>T	T	70/61/88	280 (62)	135 (60)	0.93 (0.67–1.29)	0.67
*NOS3*:c.894T>G	G	69/66/90	303 (67)	147 (65)	0.91 (0.65–1.28)	0.60

Note: 95% CI = 95% confidence interval; *—unadjusted odds ratio in the RDS and control group for allelic model; significant results were achieved when *p*-values < 0.05.

## Data Availability

Data are contained within the article.

## Abbreviations

The following abbreviations are used in this manuscript:

*NOS3*	endothelial nitric oxide synthase
*IL-10*	Interleukin-10
RDS	Respiratory distress syndrome
BPD	bronchopulmonary dysplasia
PDA	patent ductus arteriosus
CSIF	cytokine synthesis inhibitory factor
Th1	T-helper 1 cell
Th2	T-helper 2 cell
SNP	single-nucleotide polymorphism
NO	nitric oxide
FiO_2_	fraction of inspired oxygen
DNA	deoxyribonucleic acid
RNA	ribonucleic acid
PCR	polymerase chain reaction
NCBI	National Center for Biotechnology Information
MAF	minor allele frequency
qPCR	real-time polymerase chain reaction
PROMs	premature rupture of membranes
PIH	pregnancy-induced hypertension
IVF	in vitro fertilization
GDM	gestational diabetes mellitus
LISA	less invasive surfactant administration
INSURE	intubation–surfactant administration-extubation
MV	mechanical ventilation
IVH	intraventricular hemorrhage
NICU	neonatal intensive care unit
ROP	retinopathy of prematurity
HIE	hypoxic–ischemic encephalopathy
PPHN	persistent pulmonary hypertension of the newborn
OR	odds ratio
95%CI	95% confidence interval
ROC	Receiver Operating Characteristic
AUC	Area Under the Curve
NPVs	negative predictive values
PPVs	positive predictive values
